# Antioxidant and Chemopreventive Effect of Aliophen^®^ Formulation Based on Malts and Hops

**DOI:** 10.3390/antiox10010029

**Published:** 2020-12-30

**Authors:** Idolo Tedesco, Carmela Spagnuolo, Stefania Bilotto, Angelo A. Izzo, Francesca Borrelli, Daniela Rigano, Maria Russo, Fabrizio Tarricone, Gian Luigi Russo

**Affiliations:** 1 National Research Council, Institute of Food Sciences, 83100 Avellino, Italy; idolo@isa.cnr.it (I.T.); carmela.spagnuolo@isa.cnr.it (C.S.); sbilotto@gmail.com (S.B.); mrusso@isa.cnr.it (M.R.); 2 Department of Pharmacy, School of Medicine and Surgery, University of Naples Federico II, 80131 Naples, Italy; aaizzo@unina.it (A.A.I.); franborr@unina.it (F.B.); daniela.rigano@unina.it (D.R.); 3 ALIOPHARM S.r.l., 20124 Milan, Italy; fabrizio@aliopharm.com

**Keywords:** alcohol-free beer, polyphenols, antioxidant, colon cancer, chemoprevention

## Abstract

Experimental and clinical studies evidenced the health effects of moderate consumption of beer, mainly due to the presence of bioactive compounds, such as polyphenols, vitamins, or fibers. To exploit the potential beneficial effect on health and in disease prevention of these compounds, a new beverage based on barley malts and hops named Aliophen^®^ has been designed, through a patented production process, with a high total polyphenolic amount compared to alcohol-free beer and similar to the one present in light and dark beers. In the present study, the antioxidant activity of Aliophen^®^ against low-density lipoprotein (LDL) oxidation and its ability to protect erythrocytes from hemolysis have been characterized. Moreover, the chemopreventive effect of Aliophen^®^ against colon cancer has been assessed, employing a mouse model of chemically induced carcinogenesis using azoxymethane (AOM). Data obtained showed that Aliophen at a low dose (3 mg/kg) inhibited the formation of preneoplastic lesions, polyps, and tumors. At higher doses (300 mg/kg) the protective effect was measured in the first phase of the onset of cancer. The antioxidant properties of Aliophen^®^ were also observed in AOM-treated mice where it increased the serum antioxidant capacity. Based on the data presented, Aliophen^®^ can exert promising health effects, including an anticancer capacity presumably associated with its antioxidant properties.

## 1. Introduction

In the last decades, scientists dedicated many efforts to study the effects on human health of alcoholic beverage consumption, such as beer and wine, both at high and moderate intake [1,2]. High alcohol consumption is associated with harmful effects [3,4]. Among these, it is considered an important risk factor for several cancers (30 g/day), including colorectal cancer (CRC) [5]. Some studies reported that heavy (≥2 drinks/day) beer consumption may be associated with increased CRC risk [5,6]. Although data obtained so far are not conclusive, some studies indicate a positive role of moderate beer drinking (1 drink/day, about 12 g of ethanol) against mortality for any cause [7]. However, the health effects of a moderate alcohol intake are not attributable to a single factor. Multiple pieces of evidence suggest a J-shaped relationship between alcohol consumption and total mortality [8,9]. This response, which was thought to be related to the amount of alcohol consumed, is more complex; in fact, several human trials comparing spirits and fermented beverages showed different beneficial effects when similar levels of alcohol were consumed [10,11,12]. In parallel, animal studies reported that beer, but not ethanol, reduced colorectal tumorigenesis [13,14]. Therefore, it is plausible that other factors need to be considered to explain the beneficial health effects, such as the content in polyphenols, vitamins, or fibers. Recently, a large evidence-based review on the effects of beer on human health concluded that, although heavy consumption is associated with harmful effects, evidence exists that “…for no harm of moderate beer consumption for major chronic conditions and some benefit against cardiovascular disease” [15]. The international panel of experts, who authored this consensus document, also evoked a J-shaped relationship between beer intake and cardiovascular risk and identified in the presence of polyphenols part of the protective effects of beer [15]. The same view has been shared by a very recent meta-analysis on the effects of beer consumption on cardiovascular health, where the authors concluded that moderate beer consumption is beneficial in preventing dyslipidemia and in vasodilation improvement [16]. This meta-analysis highlighted an important limitation of many studies that compared the effects of beer to alcohol-free beers, attributing largely to the alcohol presence the beneficial outcomes of beer consumption in spite of its polyphenolic content, neglecting that the dealcoholization process causes a significant loss of non-alcoholic compounds, including polyphenols [17]. In fact, the polyphenol content of beer versus alcohol-free beer differs in terms of total quantity and quality of the different compounds [16]. Generally, due to the dealcoholization process, the latter contains a lower concentration of bioactive compounds compared to regular and dark beers (e.g., 120 mg/L total polyphenols vs 280–520 mg/L) [18].

Beer is brewed from natural ingredients, mainly malts, and hops, rich in amino acids, vitamins, mineral salts, and micronutrients, such as polyphenols [19]. These compounds are secondary metabolites of plants and can directly influence the sensorial characteristics of beer, providing astringency and a bitter flavor [20]. The quality and concentration of the polyphenolic compounds depend on the beer style (lager, pilsner, black and dark) and are related to the type of raw material used (grains, hop) [21]. The main structural classes of beer polyphenols include simple phenols, derivatives of benzoic acid and cinnamic acid, coumarins, catechins, proanthocyanidins. About 30% of the beer polyphenols come from hops and 70% from malt. Hops (*Humulus lupulus*, L.) are a rich source of phenolic compounds, and dried hop cones contain about 15% of polyphenols, mainly acid phenols, prenylated chalcones, flavonoids, catechins, and proanthocyanidins [22]. Barley contains different classes of phenolic compounds, such as flavonols, flavones, flavanones, chalcones, benzoic and cinnamic acid derivatives, proanthocyanidins, quinones. The malting process causes changes in the relative polyphenolic composition of barley; therefore, the industrial malt production may result in a significant increase of flavan-3-ols catechin and epicatechin and on the synthesis of sinapinic acids [22]. It is of interest the observation that melatonin is present in beers in the range 0.2–0.7 nM, independently from the alcohol content, and its presence contributed increasing the serum antioxidant capacity in healthy subjects [23].

Based on these observations, as a general preventive strategy of dietary patterns, scientists explored the biological activities of dealcoholated beverages. While some studies have shown beneficial cardio-protective effects of alcohol-free beer in men with high cardiovascular risks and in postmenopausal women [10,24], no clear evidence has been reported on the relationship between alcohol-free beer and cancer incidence. However, these studies suffer from an important confounding factor due to the absence of ethanol, which, *per se*, represents the principal carcinogenic element in alcoholic beverages. Another important issue regards the efficacy of beer polyphenols, at nutritional doses, against cancer development and progression especially those of the gastrointestinal tract [25,26].

CRC is one of the leading causes of mortality and morbidity worldwide and age, genetic, obesity, diabetes, sedentary lifestyle, low-fiber diet are important risk factors in tumor carcinogenesis [27]. The adenocarcinoma sequence, known since the 1980s, consists of the transformation of normal colorectal epithelium to an adenoma (adenomatous polyps) and ultimately to an invasive and metastatic tumor (carcinoma). The most relevant genetic changes are the activation of proto-oncogenes and inactivation of at least three tumor suppression genes: Adenomatous Polyposis Coli (APC), p53 and Loss of Heterozygosity (LOH) [28,29]. Studies in vitro and in animal models demonstrated the anticancer effect of beer polyphenols. For example, the flavonol quercetin reduces the proliferation of the Colo-205 cell line by downregulating RAS p21 protein activator 1 gene [30] and ferulic acid displays inhibition of CRC progression, acting on cells adhesion and migration mechanisms [31]. The benzoic acid derivative, gallic acid, acts on CRC cells by upregulating pro-apoptotic Bax and downregulating the anti-apoptotic Bcl-2 [32] and the chalcone xanthohumol inhibits colorectal cancer cells (HCT116 cells) downregulating the hexokinases II-mediated glycolysis [33]. In vivo studies on CRC animal models have shown that chlorogenic acid has chemopreventive effects [34] and the treatment of mice with 1% epigallocatechin-3-gallate (EGCG) causes a significant reduction in the average number of aberrant crypt foci (ACF) [35]. Quercetin (10 g/kg of diet), but not rutin (40 g/kg of diet), reduces colorectal carcinogenesis in rats treated with azoxymethane (AOM) [36]. The reduction of prostaglandin E2 (PGE2) levels has been evidenced following isohumulones treatment in male Fischer 344 rats, demonstrating their chemopreventive effects on ACF formation [37]. Finally, four commercial beers (pilsner, black, and stout) demonstrated inhibitory effects against different heterocyclic amine (HCAs)-induced ACF in rat colon, revealing a significant reduction of the risk of carcinogenesis caused by foodborne carcinogens [38].

More controversial is the study of the cause-effect relationship between the antioxidant capacity of beer polyphenols and their biological effects. A detailed characterization of potential anticancer phenolic compounds present in beers have been published in recent years [10,39] and a large part of them, individually, possesses the property to scavenge ROS (reactive oxygen species) in in vitro assays or in cell culture conditions. However, it is very difficult to demonstrate that being a phenolic antioxidant is the primary condition to favor the arrest or delay of cell proliferation in malignant cells. The opposite situation is also debated, i.e., the possibility that antioxidants favor cancer progression, as we reviewed and critically commented recently [40]. Undoubtedly, the antioxidant effects of natural phenols and their cellular derivatives in modulating the metabolism of carcinogens by regulating phase 1 metabolic enzymes and phase 2 detoxifying enzymes in addition to their anti-inflammatory properties cannot be neglected when the functional characterization of a novel mixture of phenols is undertaken.

The aim of the present work is to characterize the antioxidant effects of a new formulation based on malts and hops, named Aliophen^®^, in multiple cellular models and to assess its anticancer properties in an animal model of colon carcinogenesis. We would like to clearly state that the liquid form of Aliophen^®^, from a manufacturing and product market point of view, cannot be defined as a “beer” or “alcohol-free beer”, but, possibly, as an innovative “beverage”. However, Aliophen^®^ derived from a patented manipulation of malts and hops, the same base ingredients of beers, and contains bioactive polyphenols largely present also in beers (see Results section); therefore, our data on Aliophen^®^ bioactive properties have been discussed in comparison with those of beer to highlight similarities and differences between these two beverages and facilitate readers’ understanding.

## 2. Materials and Methods

### 2.1. Chemicals

Phosphate buffer saline (PBS) was purchased from Life Technologies (Monza, Italy), Azoxymethane (AOM), Folin-Ciocalteau’s phenol reagent, quercetin, 2.2-diphenyl-1-picrylhydrazyl, methylene blue, xylenol orange, ferrous ammonium sulfate, 2.6-di-tert-butyl-4-methylphenol (BHT), low-density lipoprotein (LDL), copper (II) sulfate, the phenolic standards used for the HPLC identification and quantification of phenolic acids were all purchased from Sigma-Aldrich (Milan, Italy). Solvents and reagents used for the preparation of mobile phases and stock solutions were also purchased from Sigma-Aldrich: water (Chromasolv^®^, for HPLC), acetonitrile (Chromasolv^®^, for HPLC reagent grade ≥ 99.9%), and formic acid (reagent grade ≥95%). Sodium carbonate, hydrogen peroxide (H_2_O_2_), sodium hypochlorite (HClO) were from Carlo Erba (Milan, Italy). All other chemicals used were of highest purity grade.

### 2.2. Aliophen^®^ Preparation

**Figure 1:**
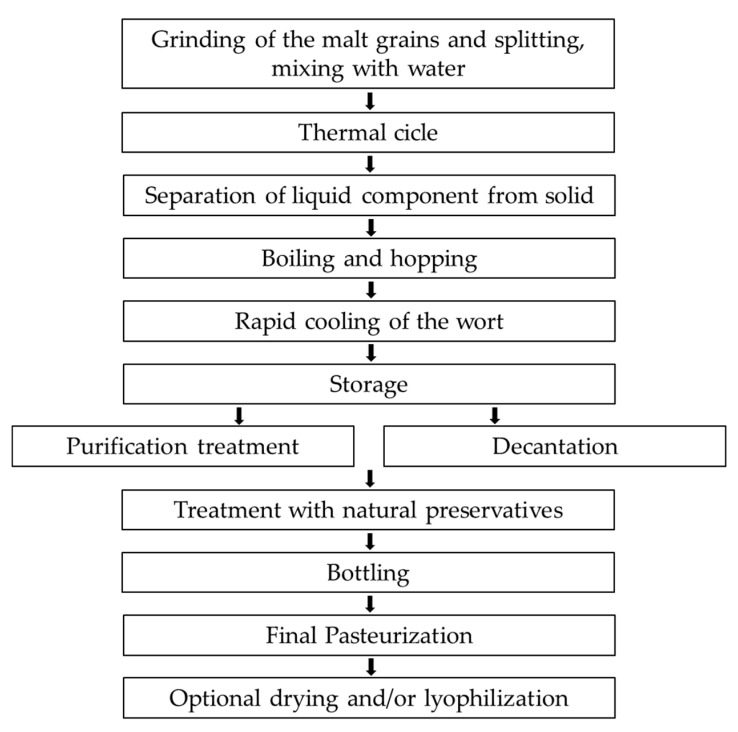
The block diagram shows the key step in the production of Aliophen^®^ (see text for description).

The trademark name Aliophen^®^ indicates a patented formulation prepared in liquid and powder forms from selected barley malts and hops. This method does not add yeasts; therefore, fermentation is not induced. The need to create an innovative polyphenolic formulation derives from the fact that normal cooking processes (like in the production of beer) or toasting (like in the production of coffee) cause a depletion of the polyphenol content naturally found in the original foodstuff. The same process takes place in the production of alcohol-free beers due to the dealcoholization methods. The method applied to Aliophen^®^ production aimed at preserving the highest quantity of natural polyphenols present in barley malts and hops avoiding fermentation and, consequently, obtain an alcohol-free product. The block diagram in Figure 1 shows the key steps of the production method. Shortly, malt grains were ground and splat into 2 portions, 20% and 80%. Each of the two fractions was mixed with water to obtain an A and a B mixture: The A mixture being prepared with the 20% portion to obtain a mixture of the malt in water at a final concentration between 9.5% and 20%. The B mixture being prepared with the 80% portion to obtain a mixture of the malt in water at a final concentration between 33% and 60%. The design of the thermal cycle was essential to obtain the desired preparation. It consisted of a first phase and a second phase, in which the first phase applies to the 20% portion (A mixture) and the second phase during which B mixture is added to the A mixture. After thermal cycles, the liquid component was separated from the solid component and boiled; then, hops were added. The wort was cooled and stored.

This method allows the obtainment of formulations in liquid (L-Aliophen^®^), powder (P-Aliophen^®^), dry (D-Aliophen^®^), and lyophilized forms. P-Aliophen^®^ was obtained using an industrial method consisting of a spray drying process that ensures a low-risk of microbiological contamination. The drying phase was conducted at high temperature but for a very short time, reducing the thermal stress on the product. The powders obtained possessed a low granulometric dispersion and a low hygroscopicity.

We also determined the nutritional composition of P-Aliophen^®^ measured by a commercial service (Chelab S.r.l., Resana, TV, Italy) using standard methods. As expected, a high concentration of sugars was present consisting essentially of about 50% of maltose (38.7 ± 3.0 g/100 g) and include sucrose, fructose, and glucose in a range of concentration between 1–9 g/100 g. Finally, P-Aliophen^®^ provides 373 ± 2 kcal/100 g (data not shown).

### 2.3. Analysis of Total Phenolic Content and Antioxidant Capacity

To perform the biochemical and biological determination on commercial beers, aliquots of each of them (1 mL) were lyophilized to remove alcohol and suspended in the same initial volume using PBS.

Total phenols in Aliophen^®^ preparations and in the lyophilized commercial beers were determined according to the Folin−Ciocalteu procedure [41]. In the case of P-Aliophen^®^ and D-Aliophen^®^, the dry material was resuspended in a volume of distilled water to reconstitute the same concentration, weight/volume, of L-Aliophen^®^, i.e., 125 mg/mL. Briefly, 5 µL of samples were mixed in an aqueous solution with Folin–Ciocalteu’s phenol reagent (5%) and sodium carbonate (2%); the mixed solution was incubated for 2 h at room temperature. The sample absorbance was measured at 760 nm and the results were expressed as equivalent of quercetin (EqQ). All measurements were carried out in triplicate [42].

Antioxidant activity was evaluated by DPPH radical scavenging assay. The scavenging capacity of beverages for the stable free radical 2.2-diphenyl-1-picrylhydrazyl (DPPH) was performed as previously [43,44] with some modifications. Samples (10 µL) were mixed to 100 µL of a methanolic solution of DPPH, vortexed for 15 s and incubated at room temperature for 30 min, before determination of absorbance at 517 nm. The antioxidant power has been indicated as the percentage of antioxidant activity (AA%) calculated following the equation: (A0 − As)/A0 × 100, where As indicates the sample absorbance and A0 the blank (absorbance of DPPH solution alone).

### 2.4. Extraction of Phenolic Compounds, Identification and Quantification

Samples (50 mL) were extracted with diethyl ether (30 mL) followed by ethyl acetate (30 mL), three times each solvent. Samples were then centrifuged (3000× *g*, 10 min) and supernatants collected. The EtOAc fractions were combined, dried with anhydrous sodium sulfate, filtered (Whatman filter, Sigma-Aldrich, Milan, Italy), and evaporated to dryness. The residue was re-dissolved in 2 mL of methanol and filtered through a 0.45 μm membrane filter (Millipore, Milan, Italy).

The main phenols were assessed by HPLC/diode-array detector (DAD) analysis by comparing their spectra and retention times with those of commercial standards. The sample (20 µL) was directly injected after filtration through a 0.45 µm membrane filter. Analyses were run on an HPLC system Jasco Extrema LC-4000 system (Jasco Inc., Easton, MD, USA) fitted with an autosampler, a binary solvent pump, and a DAD. The separation and quantification were achieved using Synergy Polar-RP C18 column (250 × 4.6 mm I.D., 5 µm particle size, Phenomenex, Torrance, CA, USA) preceded by a Polar RP security guard cartridge. The mobile phase consisted of 0.1% formic acid in water (*v*/*v*) (A) and acetonitrile (B). Injection volume was 20 µL and the flow rate was kept at 1 mL/min. Elution was performed according to the following conditions: 0–2 min, 90% (A), 2–17 min, 40% (A), 17–22 min, 40% (A), 22–28 min, 90% (A), 28–33 min, 90% (A). Quantification was performed with standard curves of external standards generated by plotting HPLC peak areas against the concentrations (mg/L) (r^2^ > 0.99). Quantitative analyses were based on external standards calibration curves [5-*O*-chlorogenic acid (5-CQA, *y* = 168.475*x* + 106.978), caffeic acid (*y* = 120.345*x* + 238.646), epicatechin (*y* = 248.360*x* + 545.689), *p*-hydroxybenzoic acid (*y* = 220.537*x* + 137.996). Based on structural skeleton similarity, caffeic acid was used for the hydroxycinnamoyl derivatives and *p*-hydroxybenzoic acid for the hydroxybenzoic acid derivatives. Results were expressed as milligrams per liter of Aliophen^®^ (mg/L). The same chromatographic conditions were applied to an HP1100 HPLC system (Agilent, Santa Clara, CA, USA) coupled to a PE-Sciex API-2000 triple-quadruple mass spectrometer (MS) (Warrington, Cheshire, UK) equipped with a turbo-spray (TSI) source as described [45].

### 2.5. Low-Density Lipoprotein Oxidation

Low-Density Lipoprotein oxidation (Ox-LDL) levels were measured as described previously [42], with some modifications, using CuSO_4_ as the oxidant agent. Briefly, 100 µg of commercial LDL proteins were incubated with or without D-Aliophen^®^ (0.25 mg/mL *w*/*v*) for 15 min. Subsequently, CuSO_4_ (50 µM) was added at 37 °C and incubated for 16 h. Finally, to determine the oxidation status the ferrous oxidation-xylenol orange (FOX) assay was performed. The assay is based on the oxidation of the Fe^2+^ that oxidized xylenol orange to a product that adsorbed at 560 nm. Thus, the FOX reagent was added for 30 min at room temperature before detecting the oxidation spectrophotometrically. Values were expressed as micromolar of H_2_O_2_ equivalents.

### 2.6. Erythrocytes Isolation and Hemolysis Assay

Peripheral blood samples from three anonymous healthy donors were provided by the Blood Donation Centre at the Onco-Hematology Department of S.G. Moscati Hospital (Avellino, Italy) with informed consent. Whole blood samples were collected in EDTA-treated tubes. Erythrocytes were isolated by centrifugation at 2000× *g* for 15 min to remove plasma, platelets, and buffy coat and washed several times in PBS. Finally, erythrocyte pellets were diluted in PBS and aliquots of 1 × 10^5^ cells/µL were used for the experiments. Hypochlorous acid (HClO) was used as the hemolytic agent [46]. The erythrocytes were pre-treated with D-Aliophen^®^ (1 mg/mL *w*/*v*) for 15 min and subsequently incubated with 0.3 mM HClO for 45 min at 37 °C. After incubation, all samples were centrifuged at 2000× *g* for 2 min and the percent of hemolysis was determined at 540 nm.

### 2.7. Cell Culture and Viability

The human promyelocytic leukemia cell line, HL-60 [47], was cultured in RPMI medium, supplemented with 10% fetal bovine serum (FBS), 1% L-glutamine, 1% penicillin, and 1% streptomycin, at 37 °C in a 5% CO_2_ humidified atmosphere. Cell density in the 48 multiwell plates was 2.5 × 10^5^/mL. Cells were incubated for 48 h in a medium added with 10 µL of diluted 1:10 Aliophen^®^ or lyophilized beers. CyQuant viability assay was performed to quantify the number of living cells using a nuclear dye that selectively binds to nucleic acids, emitting fluorescence [48]. According to the manufacturer’s protocol, CyQuant mixture, consisting of CyQuant nuclear stain and background suppressor, was added to the culture medium at the end of the treatment and incubated for 1 h at 37 °C. Fluorescence was measured using a microplate reader (Synergy HT BioTek, Milan, Italy; excitation 485 nm and emission 530 nm), and the results were expressed as a percentage of fluorescence compared to the untreated control. Microphotographs of fluorescent cells were made using an invertoscope Axiovert 200M Zeiss (Zeiss, Arese, MI, Italy).

### 2.8. Animal Treatment and Evaluations of Pre-Neoplastic Lesions, Polyps and Tumors

Experiments were performed on male ICR mice (Harlan, Italy, S. Pietro al Natisone UD, Italy) weighing 30–35 g. Animals were left in standard conditions (temperature 23 ± 2 °C; humidity 60%, free access to water and food). All experiments complied with the Italian D.L. no. 116 of 27 January 1992 and associated guidelines in the European Communities Council Directive of 24 November 1986 (86/609/ECC) and its subsequent amendments (2010/63/UE). The study was approved by the Italian Ministry of Health (identification code: 1101/2015). Mice were randomly divided into seven groups (10 mice/group) and treated as follows: group 1 with vehicles; group 2 with 300 mg/kg of D-Aliophen^®^; group 3 with 40 mg/kg AOM plus the vehicle used to solubilize the extract; groups from 4 to 7 were treated with AOM plus 3, 30, 300 mg/kg D-Aliophen^®^, respectively. AOM was administered intraperitoneally (IP) in 4 doses of 10 mg/kg at the beginning of the first, second, third and fourth week. D-Aliophen^®^ was dried at 45–50 °C for 24 h and solubilized with spring water and different doses were prepared (3–300 mg/kg) before administration orally (*per os*) daily (5 times a week) for 14 weeks starting one week before the first administration of the AOM (see Section 3.3.2). The control group received an equal volume of vehicles (spring water, 5 mL/kg *per os*; physiological 5 mL/kg IP). At the end of the treatment, all animals were euthanized by asphyxiation with CO_2_, and the colon and blood were taken. Based on previous laboratory experience, the dose of AOM and time of treatment employed in this study were sufficient to guarantee the formation of a significant number of early pre-neoplastic lesions, polyps and tumors.

For the evaluation of early pre-neoplastic lesions (aberrant crypt foci, ACF), polyps, and tumors the colon of mice was rapidly removed after killing, washed with saline, opened longitudinally, laid flat on a polystyrene board, and fixed with 10% buffered formalin solution. Subsequently, the colon was divided into 3 equal segments and stained with 0.2% methylene blue in saline. The colons of mice were examined as previously described [49], using an optical microscope (Leica Microsystems) at 20× magnification. The total number of ACF, the foci with four or more aberrant crypts (ACF ≥ 4, since they are the event immediately preceding the formation of polyps), the number of polyps, and the number of cancers were considered.

### 2.9. Peroxide Levels in Animal Serum

The whole blood was collected in tubes without anticoagulant and allow the blood to clot by leaving it undisturbed at room temperature. The clot was carefully removed by centrifugation (2000× *g* for 10 min) and the resulting serum was removed. To detect the peroxide levels, 90 µL of serum were added to 10 µL of pure methanol and 900 µL of FOX reagents (1 mM xylenol orange, 2.5 mM ferrous ammonium sulfate, and 4.4 mM of BHT in methanol). After 30 min at room temperature, samples were centrifuged at maximum speed in a microfuge, and the absorbance of the supernatants was read at 560 nm. Values were expressed as micromolar of H_2_O_2_ equivalents [50].

### 2.10. Statistics

Data are expressed as the mean ± standard deviation (SD) or, to take into account also the sample size, mean ± standard error (SEM) and analyzed by Student’s *t*-test for the evaluation of the single treatment vs. the average of the control. For the experiments performed in mice, the results were analyzed by ANOVA followed by Tukey’s multiple comparisons test (GraphPad Prism version 8.0; GraphPad Software Inc., San Diego, CA, USA). In Section 3.1, the table reporting the HPLC high resolution mass spectrometry (HR MS) data of tentatively identified phenolic compounds in D-Aliophen^®^ refer to triplicate analyses for each measurement and differences between the means were evaluated with ANOVA, using the GrafPad Instat 3 (Microsoft Software, San Diego, CA, USA) statistic program. The significance of the model was evaluated by ANOVA. The significance level was fixed at 0.05 for all the statistical analyses.

## 3. Results

### 3.1. Polyphenolic Content and In Vitro Antioxidant Capacity of Aliophen^®^

The total phenol content and the in vitro antioxidant capacity of Aliophen^®^ formulations were compared to representative examples of different beers. As reported in Table 1, the polyphenol content of Aliophen^®^, measured by Folin-Ciocalteu assay, was in the range of 2000 µM EqQ (about 60 mg EqQ 100 mL), a value from 1.5 to 4-fold higher compared to the different types of commercial beers (Table 1). Data reported in Table 1 and referring to alcohol-free, regular, red, and dark beers have been elaborated from the information reported in the Phenol-Explorer database and from previous works [18,51,52]. To highlight the efficacy of the procedure leading to Aliophen^®^ formulations, it is of note that the alcohol-free beer, whose dry matter was in the same range as that measured in L-Aliophen^®^ (about 100–125 mg/mL *w*/*v*) presented a polyphenol content of about 5 times lower.

As expected, the in vitro antioxidant capacity matched with the total polyphenol contents in the different Aliophen^®^ formulations and resulted higher than beers (Table 1). We noted that in L-Aliophen^®^ the percentage of antioxidant activity was lower compared to the dry and powder formulations despite the highest value in polyphenol content. We do not have a final explanation for this observation. It may be possible that the manufacturing procedure of spry-drying or the treatment at 45–50 °C for P-Aliophen^®^ and D-Aliophen^®^, respectively, can generate new antioxidant species or remove volatile components that may influence the DPPH radical scavenging assay.

An initial characterization of the phenolic compounds present in Aliophen^®^ was performed. The types and quantity of single polyphenols shown in Table 2 appear somehow intermediate between the polyphenolic profile of light beers and the polyphenolic profile of dark beers, and clearly different from the polyphenolic profile of non-alcoholic beers according to data retrieved from Phenol-Explorer database (data not shown). It is worthwhile to note that Aliophen^®^ contains measurable quantities of chlorogenic acid, commonly not evidenced in the polyphenolic compositions of other beers (Phenol-Explorer database). If and how these differences can influence the biological activities of Aliophen^®^ will be determined in future studies.

### 3.2. Protective Effect of Aliophen^®^ Against LDL Oxidation and Erythrocytes Oxidative Damage

To characterize the antioxidant properties of Aliophen^®^ formulations in biological systems, we evaluated its capacity to reduce the LDL oxidation induced by CuSO_4_ and to protect erythrocytes from hemolysis induced by an oxidative insult.

#### 3.2.1. LDL Oxidation

**Figure 2:**
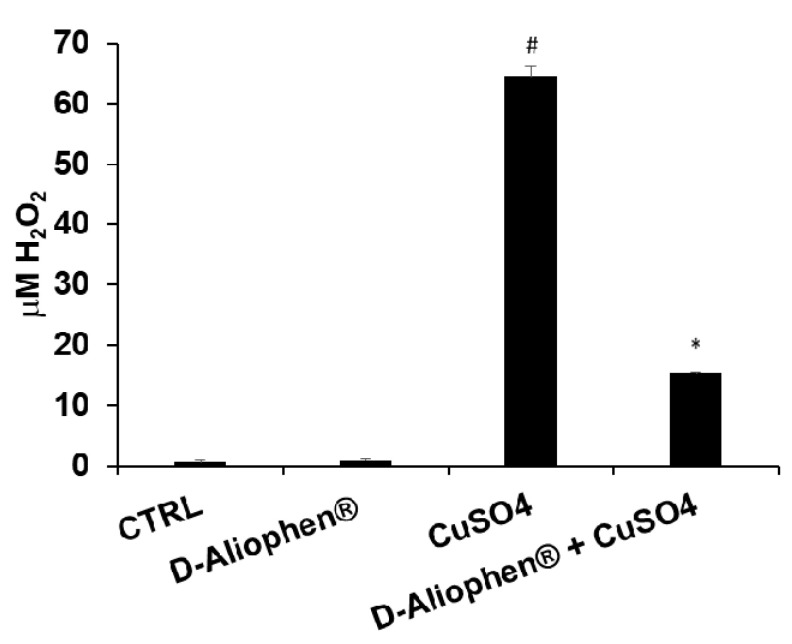
Protective effect of Aliophen^®^ against low-density lipoprotein (LDL) oxidation. LDL were incubated for 15 min in the presence of 0.25 mg/mL (*w*/*v*) of D-Aliophen^®^, before the addition of 50 µM CuSO_4_. After the treatment FOX assay was performed to determine the level of peroxide (like H_2_O_2_), produced following LDL oxidation. Bar graphs represent the mean ± SD. Symbols indicate significance: (#) *p* < 0.005 vs CTRL and (*) *p* < 0.005 vs CuSO4.

Ox-LDL are considered to play an important role in the initiation and progression of atherosclerosis, although the “oxidative modification hypothesis” of atherosclerosis is still debated and a convincing link between the plasma levels of Ox-LDL and atherosclerosis onset is still vague. However, in the framework of the function claims, the levels of Ox-LDL can represent an outcome to assess the reduction of oxidative damage to lipids [53]. Moreover, previous studies also reported that Ox-LDL may contribute to tumor development and progression and high serum levels have been positively associated with increased risk of various types of cancer, such as colorectal cancer [54,55]. To assess the protective effect of D-Aliophen^®^ against Ox-LDL, commercial LDL were treated with 50 µM CuSO_4_ resulting in about 60-fold stimulation of H_2_O_2_ concentration compared to control and D-Aliophen^®^ (Figure 2). Treating LDL for 30 min with the combination of D-Aliophen^®^ plus CuSO_4_ resulted in a strong and significant reduction in LDL oxidation (76% of reduction) compared to CuSO_4_ stimulation (Figure 2). These data suggest that Aliophen^®^ exerts a robust antioxidant effect against LDL oxidation.

#### 3.2.2. Erythrocytes Oxidative Damage

**Figure 3:**
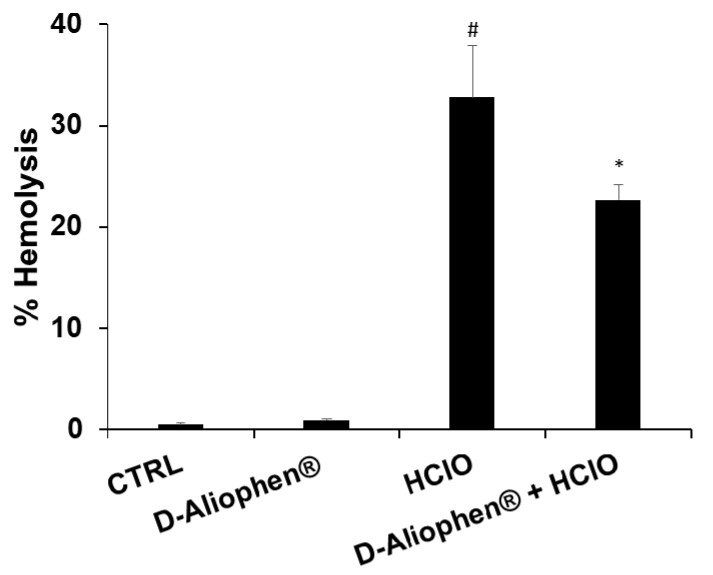
Aliophen^®^ protects erythrocytes from hemolysis induced by HClO. Erythrocytes were incubated with 1 mg/mL (*w*/*v*) of D-Aliophen^®^ for 15 min at 37 °C. Following incubation with D-Aliophen^®^, cells were stimulated with 0.3 mM of HClO for 45 min at 37 °C, the absorbance was measured at 540 nm and the percentage of hemolysis was calculated. Data are the means of samples from three subjects in duplicate ± SEM. Symbols indicate significance: (#) *p* < 0.005 vs CTRL and (*) *p* < 0.05 vs. HClO.

To verify the potential protective effects of Aliophen^®^ on the redox homeostasis in human erythrocytes, we treated RBC (red blood cells) isolated from healthy donors with HClO, which results in rapid oxidation of reduced glutathione, increases in cell osmotic fragility, and formation of transient membrane pores with the consequent hemolysis [56]. The isolated erythrocytes were stimulated with 0.3 mM HClO, a concentration in the same order of magnitude as that generated in vivo under pathological conditions [56], resulting in about 33% hemolysis (Figure 3). The pretreatment of RBC with 1 mg/mL (*w*/*v*) D-Aliophen^®^ for 45 min induced a significant, protective effect against HClO-induced hemolysis. Moreover, D-Aliophen^®^ treatment was not hemolytic *per se* (Figure 3).

### 3.3. Anticancer Effects of Aliophen^®^

In previous publications, we investigated the capacity of commercial beers to reduce cell viability in HL-60 cells, derived from a human promyelocytic leukemia cell, and correlate the antiproliferative effects with the total polyphenol content of Aliophen^®^ [51,52]. In the next paragraphs, we firstly verified if Aliophen^®^ possessed a similar in vitro antiproliferative activity and, to facilitate the comparison, we used the same cell line employed in the previous studies. Subsequently, we extended the investigation to a mice model of chemical carcinogenesis.

#### 3.3.1. Antiproliferative Effect of Aliophen^®^

**Figure 4:**
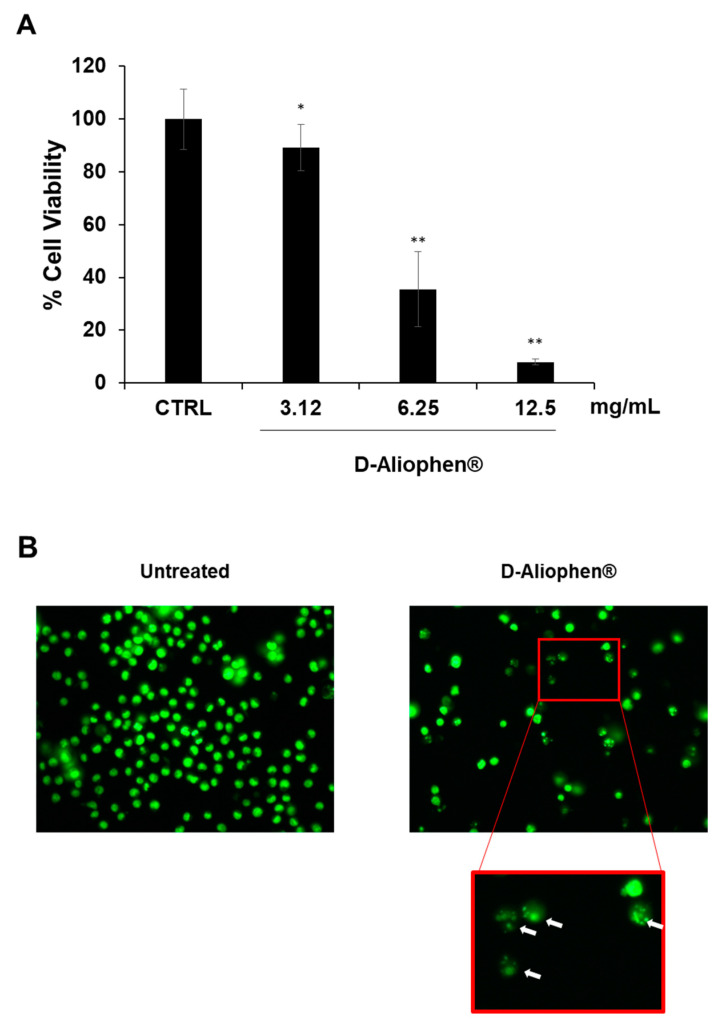
Aliophen^®^ reduces cell viability in HL-60 cells. (**A**) Cells were treated for 20 h at indicated concentration of D-Aliophen^®^, and cell viability was assessed using CyQuant as described in Materials and Methods. Bar graphs represent the mean ± SD. Symbols indicate significance vs. untreated cells (CTRL) with * *p* < 0.05 and ** *p* < 0.005. (**B**) Representative images of cells untreated (left) and treated for 20 h with 12.5 mg/mL D-Aliophen^®^ (right) and stained with CyQuant nuclear stain. Cells were visualized using a fluorescent microscopy and photographed in an FITC filter with 400× magnification. The arrows indicate the presence of apoptotic bodies.

To investigate the cytotoxic effect of D-Aliophen^®^, the dry formulation was resuspended in water to a concentration of 125 mg/mL. HL-60 cells were treated for 24 h with the indicated final concentrations of D-Aliophen^®^. The antiproliferative effect was dose-dependent and, at the highest tested concentration tested of 12.5 mg/mL, it ranged between 85–90% compared to untreated controls (Figure 4A). It is of interest that commercial beers with different polyphenol contents (Table 2**)**, in similar experimental conditions and doses, reduced HL-60 cell viability of 34–58%, linearly with the concentration of polyphenols (data not shown). Two important control experiments were also performed to confirm the specificity of the antiproliferative effects of Aliophen^®^ and their association with the presence of polyphenols. A solution of sugars resembling those present in Aliophen^®^, tested at the same final concentrations reported in Figure 4A, did not show any effect on cell viability (data not shown). Several reports suggested that inhibition of proliferation in cell lines by phenolic antioxidants could be due to the generation of hydrogen peroxide at elevated concentrations through the interaction between phenols and cell culture media components [57]. To exclude this artifactual phenomenon, we measured the ability of Aliophen^®^ preparations in the same experimental conditions described in Figure 4A to generate H_2_O_2_ in cell culture medium (without cells) by FOX assay, a method largely used to this aim [58]. As expected, no increase in the amount of H_2_O_2_ was measured (data not shown).

Moreover, observing the morphology of HL-60 treated with Aliophen^®^ (Figure 4B), it was possible to highlight the presence of numerous apoptotic bodies, suggesting that presumably the reduction in cell viability was associated with the induction of the apoptotic process (insert in Figure 4B). A similar effect of polyphenols derived from beers was previously observed with the activation of several apoptotic markers [52,59,60,61].

#### 3.3.2. Aliophen^®^ Prevents Preneoplastic Lesions, Polyps, and Tumors

To verify if the antiproliferative effects of Aliophen^®^ could be strengthened and confirmed in an animal model, L-Aliophen^®^ was dried at 45–50 °C for 24 h to obtain D-Aliophen^®^ and solubilized in spring water. Subsequently, D-Aliophen^®^ was administered to mice before and during the treatment with AOM, a potent carcinogen, that induces the transformation of colon epithelial cells into ACF, adenomas and malignant adenocarcinomas, similarly to the development of sporadic human colon cancer [62]. The thermal desiccation did not affect significantly either the total polyphenol content nor the antioxidant capacity (Table 1). The preparation of D-Aliophen^®^ used for the in vivo experiments presented a polyphenol content of 739 ± 24 µM EqQ, and an antioxidant capacity of 10.9 ± 0.35%.

A preliminary test was performed administering to the mice for 5 days D-Aliophen^®^ at 0.3, 1, 5, and 10 g/kg doses to measure acute toxicity (body weight, hair loss, death, etc.). The doses of 5 and 10 g/kg caused the death of animals after 4 days, while doses of 0.3–1 g/kg induced a reduction of body weight compared to controls (data not shown) being significant only at 1 g/kg. Based on these preliminary data, the maximum dose of D-Aliophen^®^ administered to AOM mice has been 300 mg/kg.

**Figure 5:**
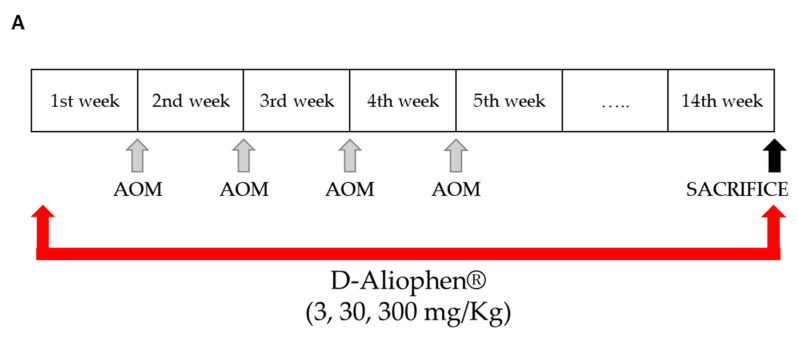

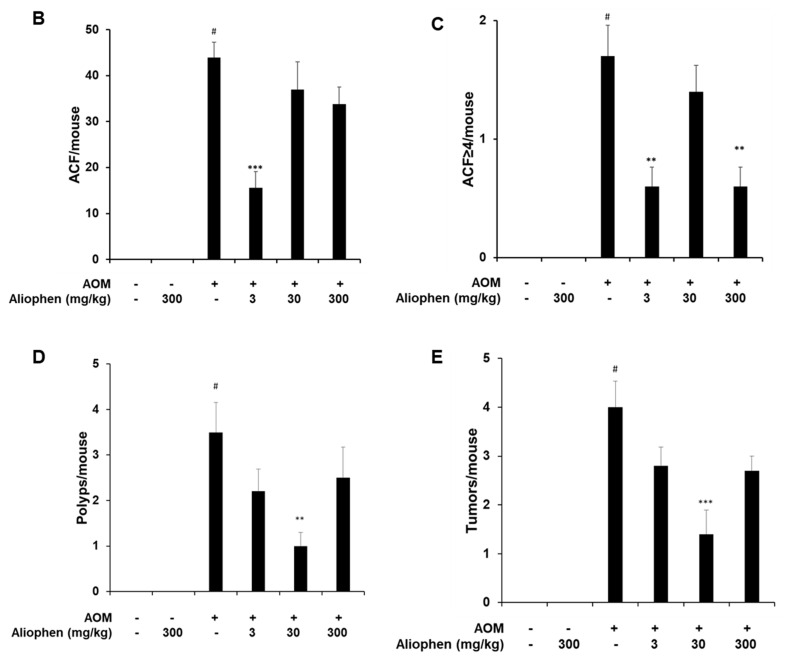
Effect of D-Aliophen^®^ against early preneoplastic lesions (aberrant crypt foci, ACF), polyps, and tumors induced in the colon of mice by azoxymethane (AOM) (**A**). Experimental design. Effect of D-Aliophen^®^ (3–300 mg/kg) on the formation of ACF (**B**), ACF ≥ 4 (**C**), polyps (**D**), and tumors (**E**) induced in the colon of mice by AOM (10 mg/kg). D-Aliophen^®^ was orally administered five times a week. The measurement of the parameters was performed 13 weeks after the first injection of AOM. Bar graphs represent the mean ± SD. Symbols indicate significance: # *p* < 0.01 or 0.001 vs. untreated, ** *p* < 0.01 and *** *p* < 0.001 vs. AOM (*n* = 5–10 mice).

To verify the preventive effect of D-Aliophen^®^ in blocking or slowing the progression of human colon cancer, the formulation was administered to mice one week before the treatment with AOM [62], which lasted 13 weeks and induced the development of ACF, polyps and tumors in the colon of mice (Figure 5A). The administration of D-Aliophen^®^ 3 mg/kg *per os*, five times a week, significantly reduced the total number of ACF/mouse (Figure 5B) and the number of ACF/mouse with four or more crypts (Figure 5C), as well as polyps (Figure 5D) and tumors (Figure 5E) induced by AOM treatment. At the dose of 30 mg/kg, D-Aliophen^®^ significantly reduced the polyps and tumor formation (Figure 5D,E) induced by AOM treatment, while no effect was observed against ACF development (Figure 5B,C). D-Aliophen^®^ at 300 g/kg showed a slight, non-significant decrease of ACF, polyps, and tumors, but only the number of ACF/mouse with four or more crypts was significantly reduced at this dosage (Figure 5C).

Overall, the results presented showed the anticancer activity of D-Aliophen^®^ in the colon-rectal district, using doses even 300-fold lower than those associated with toxicity in mice.

#### 3.3.3. In Vivo Antioxidant Activity of Aliophen^®^

**Figure 6:**
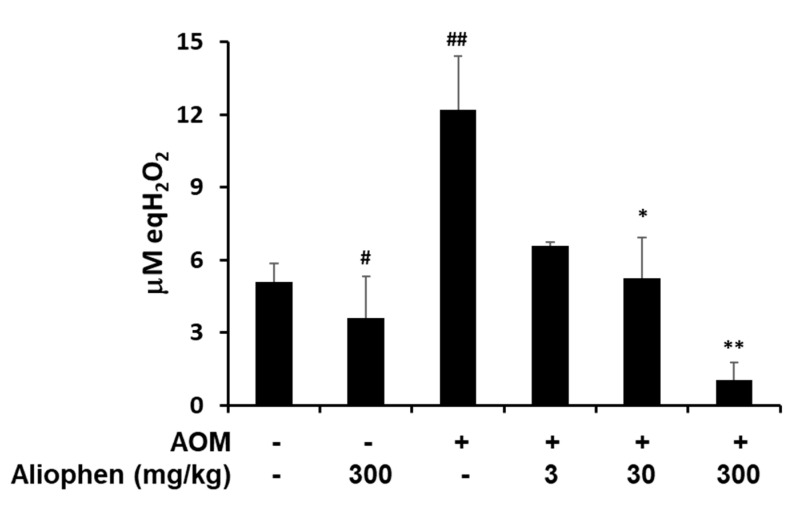
D-Aliophen^®^ reduces the oxidative stress in the serum of azoxymethane (AOM) treated mice Peroxides assay (express as equivalents of H_2_O_2_) in serum samples from mice treated with D-Aliophen^®^ (3–300 mg/kg) in the presence/absence of AOM. The samples derived from animals sacrificed at the end of the experiment. Bar graphs represent the mean ± SEM. Symbols indicate significance: # *p* < 0.01, ## *p* < 0.005 vs. untreated, * *p* < 0.05, ** *p* < 0.005 vs. AOM.

To evaluate the oxidative stress in the treated mice, we measured the peroxide concentration in the serum. As shown in Figure 6, the AOM treatment induced a strong increase of peroxide levels that were significantly reduced by D-Aliophen^®^ treatments. In particular, the peroxide levels measured in the serum of mice treated with the higher doses (30–300 g/kg + AOM) resulted lower than controls. It is also important to highlight that the treatment with 300 mg/kg of D-Aliophen^®^ alone decreased significantly the peroxide concentration compared to control, confirming the antioxidant power of the formulation under investigation.

## 4. Discussion

The world population, but also governments and industries, are increasingly interested in products that have a beneficial effect on health and can prevent chronic-degenerative diseases. This interest is reflected in the growing market of functional food ingredients that is estimated to be valued at 68.60 billion USD in 2018 and is projected to reach 94.21 billion USD by 2023, growing at a compound annual growth rate (CAGR) of 6.6%. In general, the functional foods and drinks estimated CAGR is 8% over the next eight years [63]. Similarly, the global Non-Alcoholic Beer market is valued at 4520 million USD in 2018 and will almost duplicate by the end of 2025, growing at a CAGR of 7.5% during 2019–2025 [64].

In this contest, the innovative formulation Aliophen^®^ appears extremely interesting for its chemical and biological features. The method aimed to produce Aliophen^®^ in liquid form preserves the polyphenolic fraction present in the starting material, malts and hops, maintaining its amount, 300 mg/mL, higher than in a similar beverage, i.e., alcoholic-free beers (120 mg/mL) and comparable that measured in fermented beers (280–420 mg/mL). As stated in the Introduction, Aliophen^®^, in its liquid formulation, is a beverage and not an alcohol-free beer. Therefore, the absence of fermentation can be considered an important factor that affects its polyphenol composition [65]. However, our initial data (Table 2) show that the main phenolic compounds present in Aliophen^®^ are: caffeic acid, chlorogenic acid, m-coumaric acid, *p*-hydroxybenzoic acid, sinapinic acid, epicatechin, protocatechuic acid, catechin, *p*-coumaric acid, ferulic acid, and vanillic acid. Being these phenolic compounds also present in beers (Phenol-Explorer database) and other commercial alcoholic and non-alcoholic beverages, we tend to believe that the biological effects of Aliophen^®^ can be attributed to still unknown compound(s) present in the formulation or, more likely although still speculative, to a synergistic effects of a limited number of components. On both hypotheses, we are intensively working. [66].

From a chemical and biological point of view, Aliophen^®^ appears as a very stable formulation in all its physical forms. In fact, the polyphenolic content, the antioxidant capacity, and the in vitro antiproliferative effects in HL-60 cells did not change significantly after several cycles of freezing/thawing, even after years (data not shown). This feature, together with a relatively low cost of production, at the industrial level, makes Aliophen^®^ extremely promising for applications in the nutraceutical and pharmacological sectors.

Based on the results obtained in vivo, we cannot exclude that the anticancer effect of Aliophen^®^ can depend on or be associated with its antioxidant activity. In fact, the peroxide levels measured in the serum of treated mice were significantly lowered by Aliophen^®^ treatment and this effect coincides with the reduced formation of ACF, polyps, and tumors. If the decrease of ROS is the cause of the consequence of Aliophen^®^ represents a highly debatable issue, common to other botanical extracts possessing putative antioxidant and anticancer properties. Certainly, it is well known that oxidative damage promotes tumor initiation and progression by increasing mutation rates and activating oncogenic pathways, such as impairing the Wnt/β-catenin and/or base excision repair pathways and predisposing to polyp development [67]. The oxidative DNA damage and errors in DNA polymerase favor C > T transitions in various types of adenomas and cancer, leading to a hyper-mutated phenotype of tumor cells [68]. Based on these observations, it is plausible that lowering circulating ROS can be beneficial and reduce the possibility that chemical or physical carcinogens can trigger malignant transformation. On the other hand, in the present work, the antioxidant properties of Aliophen^®^ have been further strengthened by independent experimental approaches showing that, in an in vitro assay, Aliophen^®^ clearly protects LDL from oxidation (an early event in atherosclerosis and also a phenomenon associated with increased risk of colorectal cancer) and reduces ROS-dependent hemolysis in isolated human erythrocytes. The other side of the coin reflects the opinion of influential scientists who believe that supplements based on nutritional antioxidants caused more cancers than those they prevented [69]. Recently, we critically review these controversial issues highlighting the importance of doses applied, nutritional vs pharmacological, and the cellular models selected for these studies [40]. In the case of Aliophen^®^, considering the wide range of bioactive compounds present, we can hypothesize that the anticancer effects shown in vitro and in vivo are the results of additive or synergic molecular mechanisms insisting on multiple pathways controlling cell growth not necessarily strictly related to its antioxidant properties, or even independent from them. However, the capacity of Aliophen^®^ to potentiate the serum antioxidant power can be interpreted as an additional positive effect at the systemic level, contributing to induce an antioxidant adaptive response in circulating cells and epithelial tissues that are firstly hit by carcinogens.

The results reported in the present study in a mice model of chemical carcinogenesis open the discussion to a possible pharmacological application of Aliophen^®^. The protective effect of Aliophen^®^ against colon carcinogenesis was induced at doses 100-fold lower than the toxic ones. A non-linear dose-effect emerge from our data, resulting in a lower efficacy at higher compared to lower concentrations. Considering that Aliophen^®^ contains a mixture of bioactive molecules, this phenomenon, common to other biologically active extracts, can be the result of events occurring simultaneously. Possible explanations can be: (i) the effects of a differential bioavailability and/or biotransformation of the bioactive compounds [70]; (ii) hormesis and/or adaptive response [71]; (iii) synergistic/additive/antagonistic effects of specific bioactive compounds obtained only at precise doses [72]; (iv) a combination of these and/or other events. An additional important factor to be considered is the role of the microbiota that can generate bioactive metabolites from polyphenol degradation in the intestine magnifying their intraluminal concentration [73]. Several studies have reported the relationship between polyphenols present in beer (ferulic acid, xanthohumol, catechins, epicatechins, proanthocyanidins, quercetin, and rutin) and increased growth of beneficial bacteria in gut microbiota [66].

Although we are very cautious in the exercise of extrapolating results obtained in animal models to humans, according to a recent guide for dose conversion between animals and human [74], the dose of 30 mg/kg in mice corresponds to an equivalent dose of about 146 mg in a man of 60 kg. This amount becomes 10 times less (14.6 mg/60 kg), considering the dose of 3 mg/kg that was effective in the first phase of ACF formation in mice. From this extrapolation, it appears that D-Aliophen^®^ efficiently reduced colon carcinogenesis at dosages compatible with the administration of dietary supplements in humans. In addition, considering that the Aliophen^®^ provides about 370 kcal/100 g, the putative human dose of 146 mg corresponds to a negligible number of calories.

Even if these data appear promising in cellular and animal studies, both showing the preventive effect of dietary antioxidants against cancer developing, we should not give in to easy enthusiasm and carefully consider the data. In fact, many observational studies, including case–control and cohort studies, investigating whether the use of dietary antioxidant supplements is associated with reduced risks of cancer in humans, yielded mixed results [75,76,77]. In particular, randomized controlled clinical trials of dietary antioxidant supplements for cancer prevention, that provide the strongest and most reliable evidence of the effects of a health-related intervention, found no clear evidence [75]. The failure to confirm preclinical data could be due to the differences between the effects of consuming purified chemicals or antioxidants in foods, to the different amounts used in the studies, or to the effects of metabolism on bioactive compounds [40,78].

In the future, we will finely identify and characterize polyphenols present in Aliophen^®^ and select appropriate in vitro models to identify the early cellular target(s) of the most bioactive compounds. The quantification of their cellular uptake (or those of their metabolites) will allow us to determine the minimal intracellular concentrations necessary to trigger the observed anticancer activity. Thus, considering the low toxicity, we can predict potential beneficial effects in combination therapy or in secondary cancer prevention, stimulating the design of optimized clinical trials.

## 5. Conclusions

The results reported in the present study show the antioxidant and protective effects against colon carcinogenesis of Aliophen^®^, a formulation prepared from malts and hops, the same components present in beer. The selection of barley malt varieties and hops together with the new production process represented important factors in considering the quality and the antioxidant properties of Aliophen^®^. In liquid form, Aliophen^®^ increased serum antioxidant capacity in mice, reduced in vitro LDL oxidation induced by CuSO_4_, and protected human erythrocytes from hemolysis.

Aliophen^®^, at the dose of 3 mg/kg acted both in the first phase of the onset of cancer (formation of pre-neoplastic lesions) and in the second phase (formation of polyps and tumors). Considering the data obtained, the anticancer effect exerted in AOM-treated mice could be presumably associated with its antioxidant properties. Despite the limitations of the present study, the antitumor effect of Aliophen^®^ in the murine model of carcinogenesis represents an important starting point for future studies and clinical trials of phase I-II.

## 6. Patents

Aliopharm S.r.l. has in sharing with the National Research Council of Italy the ownership of the international patent application PCT/IB2018/056283.

## Figures and Tables

**Table 1:** Polyphenolic content, of different Aliophen^®^ formulations compared to commercial beers.

Samples	Polyphenol Content (µM EqQ) **	Polyphenol Content (mg EqQ/100 mL)	AA (%) ***
L-Aliophen^®^ *	2312 ± 580	69.87 ± 17.5	25.55 ± 7.4
P-Aliophen^®^ *	1798 ± 450	54.34 ± 13.6	45.75 ± 12.2
D-Aliophen^®^ *	1879 ± 470	57.1 ± 14.2	37.3 ± 10.8
Commercial beers			
alcohol-free	480 ± 12	14.5 ± 0.40	4.0 ± 0.03
Regular	886 ± 49	26.8 ± 1.5	10.9 ± 0.3
Red	1188 ± 32	35.9 ± 1.0	11.8 ± 0.4
Dark	1356 ± 30	41.0 ± 0.9	8.5 ± 0.08

* Data reported for D-Aliophen^®^ refer to reconstitute solutions of about 125 mg/mL (*w*/*v*). ** EqQ, equivalent of quercetin. *** AA, Antioxidant Activity. Values indicate mean ± SD.

**Table 2:** High Resolution Mass Spectrometry HPLC data of tentatively identified phenolic compounds in D-Aliophen^®^.

Phenolic Compound	Molecular Formula	RT (min)	Molecular Weight	[M − H]^−^ Found (*m*/*z*)	[M − H] (*m*/*z*)	Relative Amount * (mg/L)
Protocatechuic acid	C_7_H_6_O_4_	6.5	154.0266	153.0197	153.0193	0.80 ± 0.01
Vanillic acid	C_8_H_8_O_4_	7.5	168.1467	167.0370	167.0371	1.24 ± 0.02
Catechin	C_15_H_14_O_6_	15.7	290.0790	289.0721	289.0717	2.09 ± 0.07
Chlorogenic acid	C_16_H_18_O_9_	18.2	354.0951	353.0868	353.0878	0.88 ± 0.01
*p*-Hydroxybenzoic acid	C_7_H_6_O_3_	19.5	138.0317	137.0237	137.0244	0.47 ± 0.03
Caffeic acid	C_9_H_8_O_4_	21.0	180.0423	179.0341	179.0350	0.49 ± 0.07
Epicatechin	C_15_H_14_O_6_	23.7	290.0769	289.0699	289.0696	1.45 ± 0.09
*p*-Coumaric acid	C_9_H_8_O_3_	24.4	164.0473	163.0400	163.0393	2.53 ± 0.09
*m*-Coumaric acid	C_9_H_8_O_3_	24.9	164.0473	163.0416	163.0400	0.51 ± 0.04
Sinapic acid	C_11_H_12_O_5_	28.9	224.0685	223.0604	223.0605	0.89 ± 0.03
Ferulic acid	C_10_H_10_O_4_	29.8	194.0579	193.0504	193.0505	4.93 ± 0.07

* Expressed as mean value ± SD.

## Data Availability

The data presented in this study are available on request from the corresponding author. Part of the data are not publicly available due to possible commercial exploitation.
